# Expression of SLC2A9 Isoforms in the Kidney and Their Localization in Polarized Epithelial Cells

**DOI:** 10.1371/journal.pone.0084996

**Published:** 2014-01-07

**Authors:** Toru Kimura, Michi Takahashi, Kunimasa Yan, Hiroyuki Sakurai

**Affiliations:** 1 Department of Pharmacology and Toxicology, Kyorin University School of Medicine, Mitaka, Tokyo, Japan; 2 Department of Pediatrics, Kyorin University School of Medicine, Mitaka, Tokyo, Japan; Tohoku University, Japan

## Abstract

**Background:**

Many genome-wide association studies pointed out that *SLC2A9* gene, which encodes a voltage-driven urate transporter, SLC2A9/GLUT9 (a.k.a. URATv1), as one of the most influential genes for serum urate levels. *SLC2A9* is reported to encode two splice variants: SLC2A9-S (512 amino acids) and SLC2A9-L (540 amino acids), only difference being at their N-termini. We investigated isoform-specific localization of SLC2A9 in the human kidney and role of N-terminal amino acids in differential sorting in vitro.

**Methodology/Principal Findings:**

Isoform specific antibodies against SLC2A9 were developed and human kidney sections were stained. SLC2A9-S was expressed in the apical side of the collecting duct while SLC2A9-L was expressed in the basolateral side of the proximal tubule. GFP fused SLC2A9s were expressed in MDCK cells and intracellular localization was observed. SLC2A9-S was expressed at both apical and basolateral membranes, whereas SLC2A9-L was expressed only at the basolateral membrane. Although SLC2A9-L has a putative di-leucine motif at 33th and 34th leucine, deletion of the motif or replacement of leucine did not affect its subcellular localization. When up to 16 amino acids were removed from the N-terminal of SLC2A9-S or when up to 25 amino acids were removed from the N-terminal of SLC2A9-L, there was no change in their sorting. Deletion of 20 amino acids from SLC2A9-S was not expressed in the cell. More than 30 amino acids deletion from SLC2A9-L resulted in expression at both apical and basolateral membranes as well as in the lysosome. When amino acids from 25th and 30th were changed to alanine in SLC2A9-L, expression pattern was the same as wild-type.

**Conclusions/Significance:**

SLC2A9-L was expressed in the basolateral membrane of kidney proximal tubules in humans and this isoform is likely to responsible for urate reabsorption. N-terminal amino acids unique to each isoform played an important role in protein stability and trafficking.

## Introduction

Since 2007, many genome wide association studies (GWASs) have shown that SNPs in *SLC2A9* are correlated with serum urate levels and/or gout [1], [2], [3], [4], [5]. Based on the similarity of the predicted topology, a gene product of *SLC2A9* was originally reported to be a member of the facilitative glucose transporter family and named glucose transporter (GLUT) 9 [6]. Augustin et al reported that the *SLC2A9* gene produces 2 splice variants that only differ in their N-termini and that both isoforms are expressed in the human kidney [7]. In the same paper, these splice variants are shown to be targeted to the different membrane domains when they are expressed in polarized Madin-Darby canine kidney (MDCK) cells; the longer one (GLUT9, SLC2A9-L in this paper) goes to the basolateral membrane while the shorter one (SLC2A9ΔN, SLC2A9-S in this paper) to the apical membrane. Although SLC2A9-expressing oocytes take up deoxyglucose, functional data for SLC2A9 had been scant before aforementioned GWASs.

After GWAS data were obtained, we and others have demonstrated that SLC2A9 indeed transports urate in vitro [5], [8], [9], [10]. We have shown that SLC2A9 transports urate in a voltage-dependent fashion, from negative to positive direction [8] and proposed to rename it to URATv1 (voltage –driven urate transporter 1). Subsequently loss-of-function mutations of SLC2A9 in humans are shown to cause renal hypouricemia, indicating that SLC2A9 plays a critical role in urate reabsorption in the kidney [8], [10], [11], [12], [13], [14]. Augustin et al showed that SLC2A9 is expressed in the basolateral membrane of the kidney proximal tubular cell [7] using the antibody recognizing both isoforms. Given the fact that intracellular potential is negative to the interstitial space, basolateral localization of this transporter favors urate exit from the tubular cell and is consistent with urate reabsorptive function. Another urate reabsorptive transporter, SLC22A12/URAT1, is known to be expressed at the apical membrane of the proximal tubular cells [15]. Thus, we proposed that urate is reabsorbed via SLC22A12 from the urinary space into the kidney proximal tubular cell and then out of the cell to the interstitial space via SLC2A9 [16]. Based on the differential trafficking of 2 splice variants in the polarized epithelial cell, it is likely that SLC2A9 expressed in the basolateral membrane of the kidney proximal tubule is the longer isoform. However, isoform-specific localization in the kidney has not been reported to date.

It has been originally shown that SLC2A9 transport glucose and fructose [17]. Few years later, we have shown that SLC2A9-L (a.k.a. GLUT9, GLUT9a; consists of 540 amino acids) and SLC2A9-S (a.k.a. SLC2A9ΔN, SLC2A9b; consists of 512 amino acids) transport urate in a similar manner when they are expressed in Xenopus oocytes [8]. Recently, more detailed functional characterization has been published [18]. Consistent with our data, they do not find a major difference between these 2 isoforms in transporting urate as a sole substrate. However, the fact that urate transport mediated by SLC2A9-L, but not SLC2A9-S, is modified by the presence of hexoses in/out of the cell [18] suggests that difference in N-terminal regions may affect not only their apical/basolateral sorting but also their transport function. Nonetheless, regulating apical/basolateral trafficking is important for SLC2A9 given its vectorial urate transport function. A putative di-leucine (LL) motif, which has been shown to be important for basolateral localization of many proteins in the epithelial cells, is present at N-terminal of SLC2A9-L. However, Bibee et al showed that LL sequence of SLC2A9-L is not critical for its basolateral localization in MDCK cells [19].

In this study, using isoform specific antibodies, localization of SLC2A9 isoforms in the human kidney was examined. In addition, effect of N-terminal amino acids in regulating apical/basolateral trafficking of SLC2A9 in polarized MDCK cells was investigated by constructing deletion or substitution mutants of SLC2A9 isoforms.

## Results

### Localization of SLC2A9-S and -L in Human Kidney

**Figure 1:**
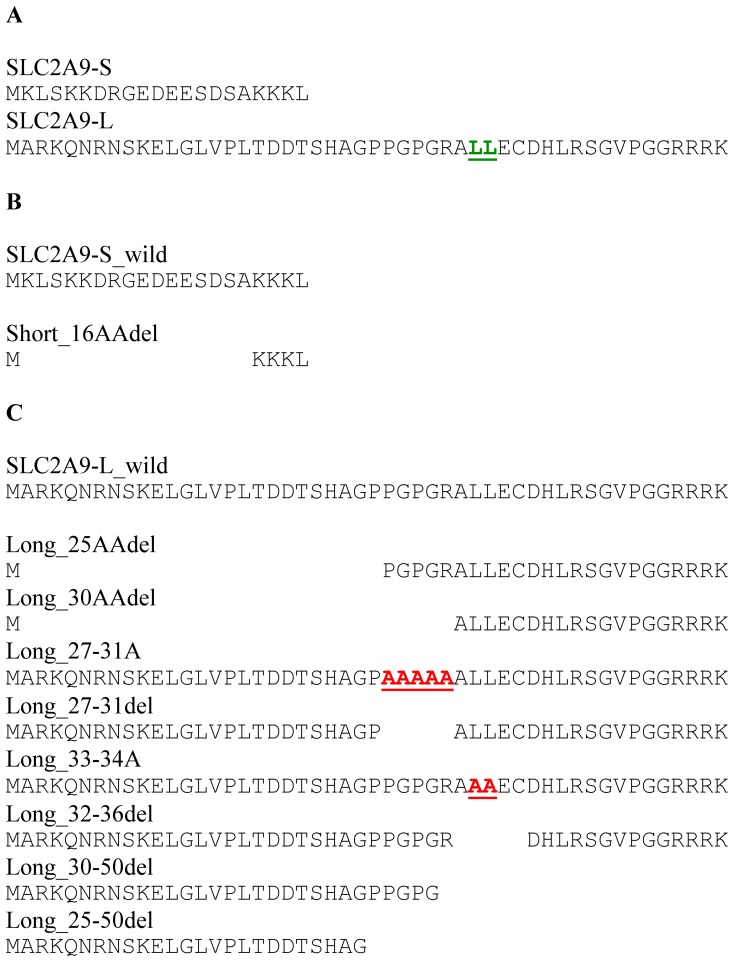
Amino acids sequences of SLC2A9 N-termini of 2 wild types and mutant constructs. A. Two isoforms, SLC2A9-S and SLC2A9-L, have different N-termini of 21 and 50 amino acids, respectively. B, C. Deleted or mutated constructs of SLC2A9-S (B) and -L (C) were shown.

**Figure 2:**
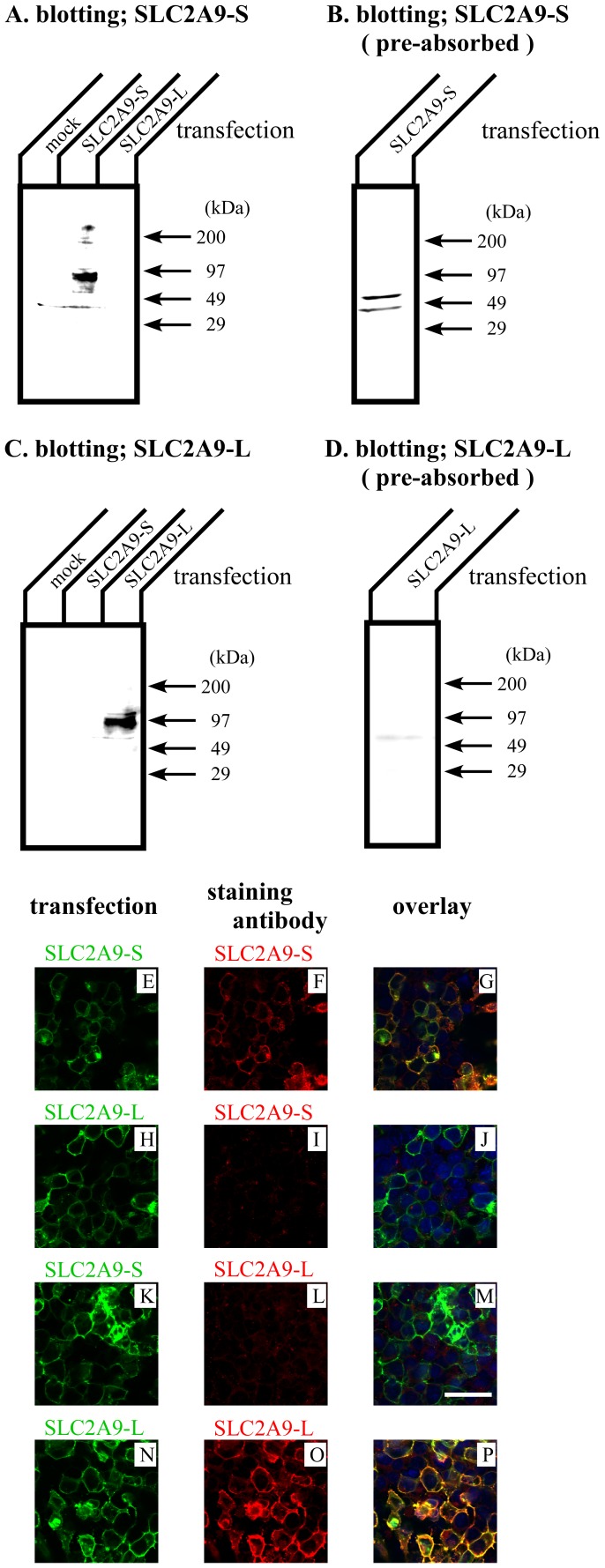
Characterization of isoform specific SLC2A9 antibodies. 30 µg of COS cell lysate transfected with mock or SLC2A9s were separated by SDS-PAGE and Western blotting was performed with anti-SLC2A9-S (A), antigen pre-absorbed anti- SLC2A9-S (B), anti-SLC2A9-L (C), or antigen pre-absorbed anti-SLC2A9-L (D) antibodies. COS cells were transfected with GFP tagged SLC2A9-S (E and K) or SLC2A9-L (H and N) and immunofluorescence was performed with anti-SLC2A9-S (F and I) or anti-SLC2A9-L (L and O) antibodies. Overlay images were shown in G, J, M and P. The scale bar of 40 µm was shown in K.

*SLC2A9* gene is reported to have 2 splice variants: short isoform (SLC2A9-S) with 512 amino acids and long isoform (SLC2A9-L) with 540 amino acids. Their C-terminal 490 residues are identical and only difference lies at their N-termini. SLC2A9-S and SLC2A9-L have different N-termini of 21 and 50 amino acids, respectively (Fig. 1A). To detect specific expression of SLC2A9-S and SLC2A9-L, antibodies against these different amino acid residues were produced. Western blotting was performed with these antibodies to check the specificity (Fig. 2A–D). COS cells were transfected with mock, SLC2A9-S and SLC2A9-L, and cell lysate was blotted with anti-SLC2A9-S (Fig. 2A) or anti-SLC2A9-L (Fig. 2C) antibodies. These antibodies specifically detected each SLC2A9 isoform without any cross reactivity. The signals corresponding to SLC2A9s were disappeared when Western blotting was performed with antibodies pre-absorbed with immunogens (Fig. 2B and 2D). Immunofluorescent staining was also used to confirm the specificity of these antibodies (Fig. 2E–P). Green fluorescence protein (GFP) tagged SLC2A9-S or SLC2A9-L was transfected into COS cells and cells were stained with anti-SLC2A9-S (Fig. 2E–J) or anti-SLC2A9-L (Fig. 2K–P) antibodies. Anti-SLC2A9-S and anti-SLC2A9-L antibodies did not recognize the COS cells expressing SLC2A9-L (Fig. 2 I) and SLC2A9-S (Fig. 2L), respectively. In contrast, anti-SLC2A9-S and anti-SLC2A9-L antibodies specifically detect COS cells transfected with SLC2A9-S (Fig. 2F) and SLC2A9-L (Fig. 2O), respectively.

**Figure 3:**
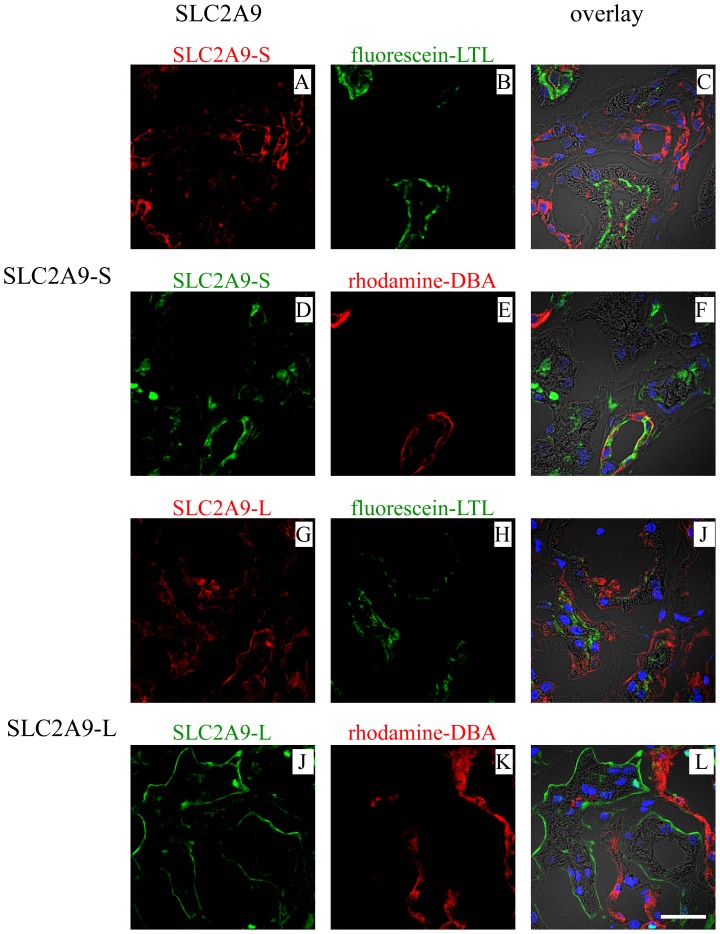
SLC2A9 expression in human kidney. SLC2A9-S (A,D) or SLC2A9-L (G,J) was stained together with fluorescein-Lotus Tetragonolobus lectin (LTL; green) (B,H) or rhodamine - Dolichos Biflorus agglutinin (DBA; red) (E,K). Overlay images staining with nucleus (blue) and with phase contrast images were shown in C, F, I and L. The scale bar of 30 µm was shown in L.

Human kidney sections were stained with these antibodies together with fluorescein-Lotus Tetragonolobus lectin (LTL) or rhodamine - Dolichos Biflorus agglutinin (DBA) (Fig. 3); LTL specifically binds to renal proximal tubules and DBA specifically binds to collecting ducts. SLC2A9-S was expressed at the apical membrane of collecting ducts, which were stained with DBA (Fig. 3D–F). In contrast, SLC2A9-L was expressed at the basolateral membrane of proximal tubules, which were positive for LTL (Fig. 3G–J). Some PECAM-1 (Platelet Endothelial Cell Adhesion Molecule-1) positive endothelial cells expressed SLC2A9-S or -L (data not shown). Only weak background signal was obtained when immunostaining was performed using these antibodies pre-incubated with their antigen peptides (data not shown).

### Expression Patterns of SLC2A9-S and -L in Polarized MDCK Cells

**Figure 4:**
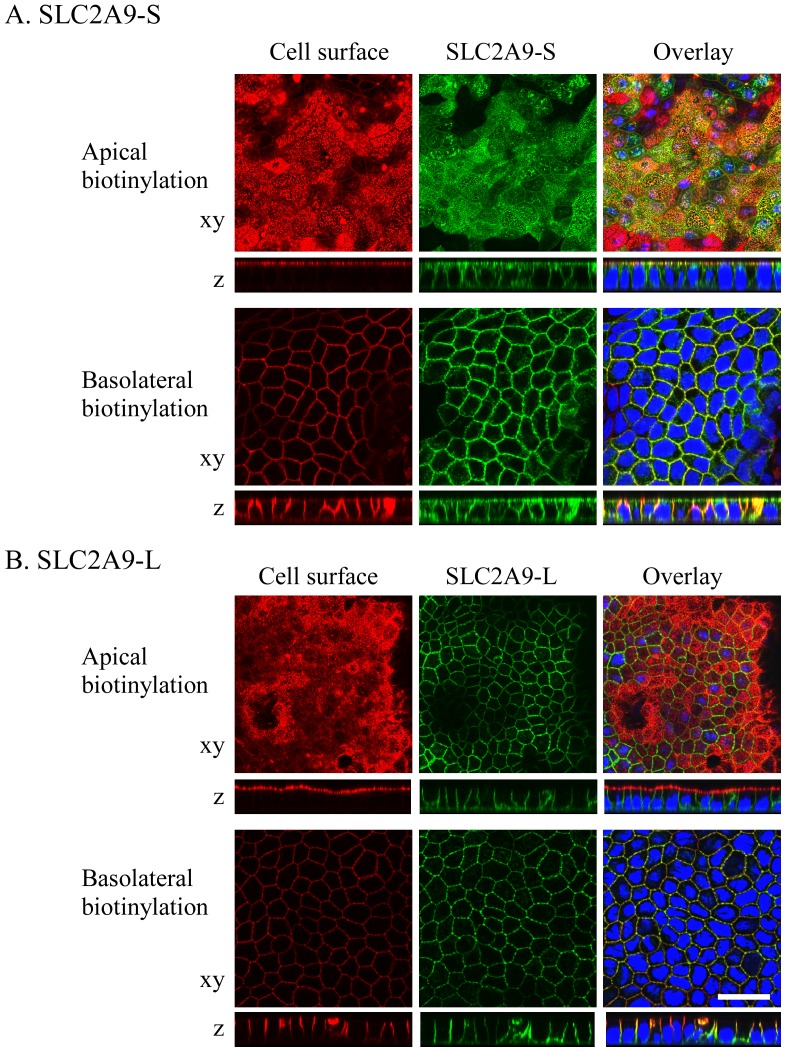
Expression of fluorescent-tagged SLC2A9 isoforms in MDCK cells. GFP-tagged (green) SLC2A9 -S (A) or -L (B) constructs were transfected into MDCK cells. Cell surface of the apical or basolateral membrane was biotinylated and visualized in red. Confocal images of xy and z axes were obtained and the white scale bar was 30 µm.

In the human kidney, SLC2A9-S was expressed at the apical membrane, whereas SLC2A9-L was expressed at the basolateral membrane in the different renal tubules. This sorting pattern is consistent with the previous report using MDCK cells [7]. However, the paper failed to report the mechanism of differential sorting of these 2 isoforms. Here, we pursued this question in detail. GFP tagged SLC2A9-S or -L was transfected into polarized MDCK cells (MDCK type II). To identify apical or basolateral membrane, cell surface was labeled with biotin. When GFP tagged SLC2A9-S was expressed in MDCK cells, it was distributed in both apical and basolateral membranes (Fig. 4A). Some part of SLC2A9-S was localized in the intracellular compartment. On the other hand, SLC2A9-L was expressed only at the basolateral membrane of MDCK cells (Fig. 4B). These results indicate that intracellular trafficking of SLC2A9 in polarized cells is determined by its N-terminal residues, only differences between SLC2A9-S and -L. Interestingly, SLC2A9-S localized differently in human renal tubule cells and cultured MDCK cells.

### Effect of Deletion of SLC2A9-S N-terminus on its Localization

**Figure 5:**
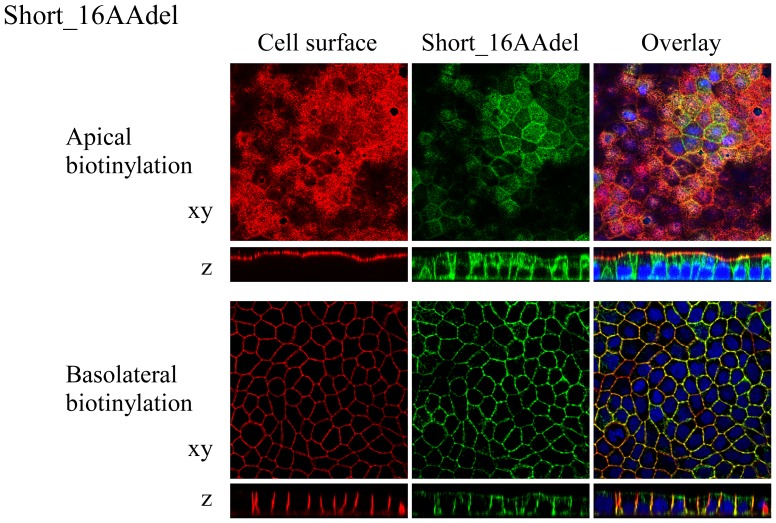
Localization of N-terminal deletion constructs of SLC2A9-S. GFP-tagged Short_16AAdel construct (green), which was deleted with N-terminal 16 amino acids from SLC2A9-S, was transfected into MDCK cells. Cell surface of the apical or basolateral membrane was biotinylated and visualized in red. Conforcal images of xy and z axes were obtained and the magnification is the same as Fig. 3.

SLC2A9-S was expressed at both apical and basolateral membranes in MDCK cells (Fig. 4A). To reveal the role of SLC2A9-S N-terminus on this expression pattern, amino acids were deleted stepwise by 4 residues and its sorting was observed in MDCK cells. Removal of the N-terminal 4∼16 amino acids from SLC2A9-S did not affect its sorting and these deletion mutants were localized both in the apical and basolateral membranes similar to SLC2A9-S wild-type. Localization of the shortest deletion mutant, Short_16AA (Fig. 1B), in MDCK cells was presented in Fig. 5. Short_16AA was expressed both in the apical and basolateral membranes. As noted with SLC2A9-S wild-type, some of Short_16AA was localized at the intracellular compartment. Removal of the N-terminal 20 amino acids unique to SLC2A9-S resulted in very low protein expression (data not shown). These results suggest that N-terminal of SLC2A9-S does not contain an apico-basolateral sorting signal recognized by MDCK cells unlike renal collecting duct cells. N-terminal amino acids unique to SLC2A9-S may be important for protein expression/stability at least in MDCK cells.

### Significance of Putative Di-leucine Motif in SLC2A9-L N-terminus on the Polarized Expression

**Figure 6:**
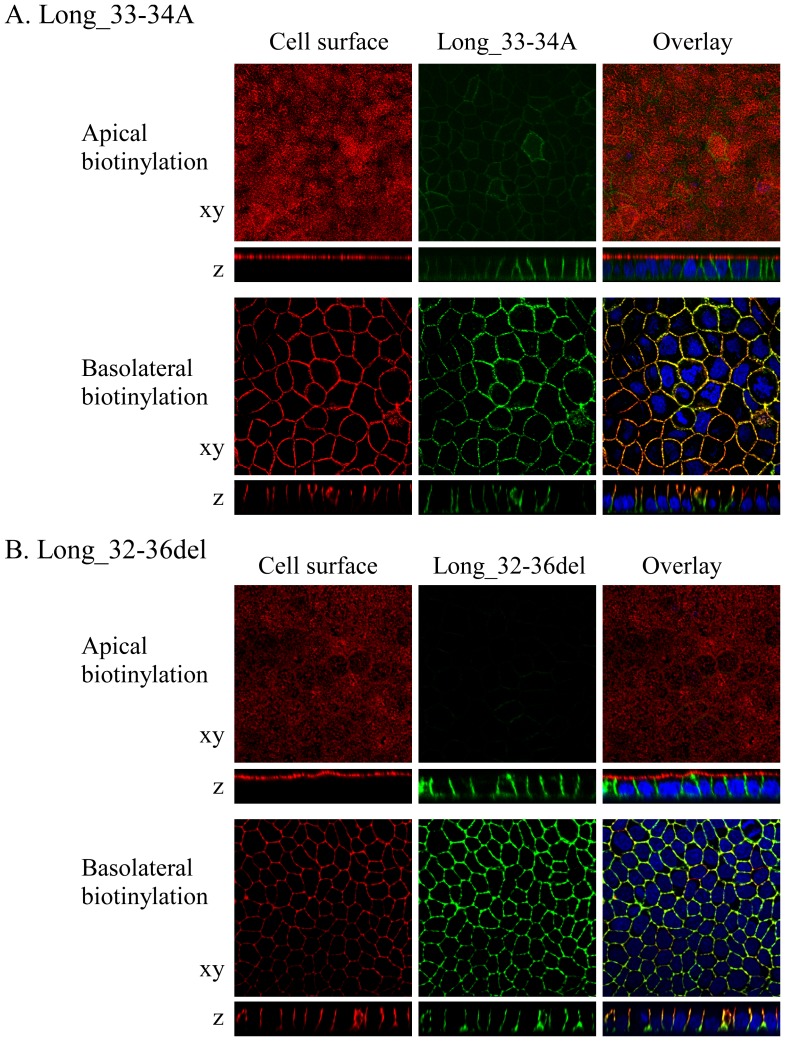
Significance of the putative di-leucine motif in SLC2A9-L on basolateral sorting. A. GFP-tagged Long_33-34AA (green), in which 33^rd^ and 34^th^ leucine-leucine was changed to alanines were expressed in MDCK cells. B. GFP-tagged Long_32-36del (green), in which 32^nd^–36^th^ amino acids including the putative di-leucine motif was deleted, were expressed in MDCK cells. Cell surface of the apical or basolateral membrane was biotinylated and visualized in red. Confocal images of xy and z axes were obtained and the magnification is the same as Fig. 3.

Di-leucine (LL) motif is often critical for trafficking of membrane proteins such as basolateral targeting and cell surface/intracellular recycling (endocytosis). SLC2A9-L has a putative di-leucine motif at 33th and 34th leucine and it may be important for basolateral sorting of SLC2A9-L. This LL is an obvious candidate that regulates basolateral targeting of SLC2A9-L in the polarized epithelial cell. To evaluate the role of the LL, 2 mutant constructs were made: Long_33-34A, in which the LL was substituted with alanine-alanine, and Long_32-36del, in which 5 amino acids around the LL were deleted. When these mutants were expressed in MDCK cells, they were targeted to the basolateral membrane similar to SLC2A9-L wild-type (Fig. 6). These results indicate that the LL sequence in SLC2A9-L does not work as a typical di-leucine motif and neither LL sequence nor its surrounding amino acids are critical for its basolateral sorting.

### Significance of SLC2A9-L N-terminus on the Polarized Expression

**Figure 7:**
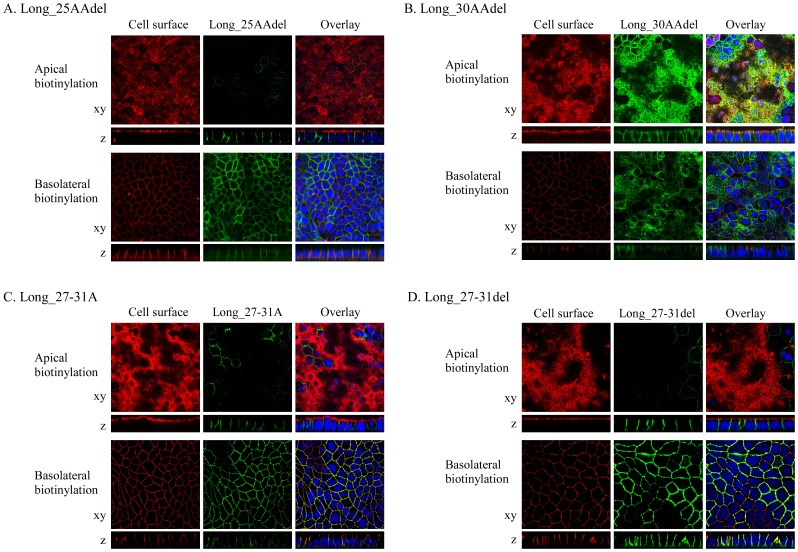
Effect of N-terminal deletion or exchange of SLC2A9-L. Long_25AAdel (A) or Long_30AAdel (B) constructs, in which N-terminal 25 or 30 amino acids were deleted from SLC2A9-L, respectively, Long_27-34A (C), in which amino acids from 27^th^ and 31st were changed to alanines, or Long_27-31del (D), in which 5 amino acids from 27^th^ and 31^st^ were deleted, were transfected into MDCK cells. These GFP-tagged constructs were visualized in green. Cell surface of the apical or basolateral membrane was biotinylated and visualized in red. Confocal images of xy and z axes were obtained and the magnification is the same as Fig. 3.

To identify the important amino acid residues of SLC2A9-L for its basolateral membrane sorting, several N-terminal deletion mutants were made (Fig. 1). N-terminus of SLC2A9-L was deleted stepwise by 5 amino acids and those mutants were transfected into MDCK cells. When 5, 10, 15, 20 or 25 amino acids were removed from SLC2A9-L N- terminal, these mutants went to the basolateral membrane similar to SLC2A9-L wild-type. Localization of Long_25AAdel, in which 25 amino acids from SLC2A9-L N-terminal were removed, was shown in Fig. 7A. Removal of the N-terminal 30 amino acids from SLC2A9-L (Long_30AAdel), however, resulted in both apical and basolateral membrane expression (Fig. 7B) similar to the localization of SLC2A9-S. Based on these results, amino acids 27^th^–31^st^ of SLC2A9-L (PGPGR) appeared to be important in basolateral targeting of this transporter. To test this hypothesis, alanine substitution and deletion mutant constructs were made. To our surprise, Long_27-31A mutant, in which 27^th^–31^st^ amino acids were replaced with alanine, was expressed exclusively at the basolateral membrane (Fig. 7C). Deletion of those five amino acids (Long_27-31 del) did not affect its basolateral localization (Fig. 7D), either.

**Figure 8:**
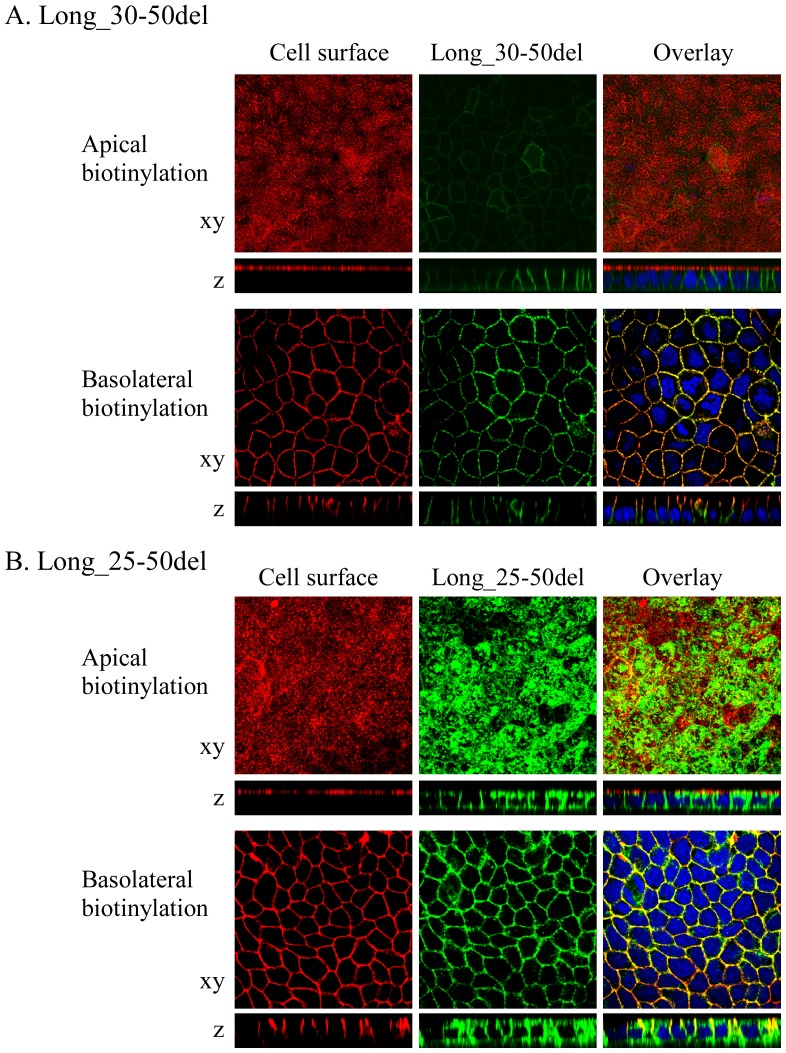
Effect of N-terminal deletion of SLC2A9-L near the transmembrane side. GFP-tagged Long_30-50del (A), in which amino acids from 30th to 50th were deleted, or GFP-tagged Long_25-50del (B), in which amino acids from 25th to 50th were deleted from SLC2A9-L were expressed in MDCK cells. These constructs were visualized in green. Cell surface of the apical or basolateral membrane was biotinylated and visualized in red. Conforcal images of xy and z axes were obtained and the magnification is the same as Fig. 3.

When 21 amino acids from 30th to 50th were deleted, the deletion construct, Long_30-50del, was expressed only at the basolateral membrane similar to SLC2A9-L wild-type (Fig. 8A). On the other hand, when 26 amino acids from 25th to 50th were deleted, the deletion construct, Long_25-50del, was localized both at the apical and basolateral membranes (Fig. 8B). The difference between Long_30-50del and Long_25-50del is PPGPG sequence (26th–30th), which was also removed in Long_30AAdel, which was localized both in the apical and the basolateral membrane (Fig. 7B) However we have already shown that PGPGR (27th–31st) was dispensable for basolateral trafficking of SLC2A9-L (Fig. 7, C and D). It is possible to argue that 26^th^ amino acid proline is important, but N-terminal deletion mutant upto the 26^th^ proline, Long_25AAdel, was sorted correctly to the basolateral membrane (Fig. 7A). Taken together, N-terminal amino acids unique to SLC2A9-L was important in its basolateral membrane targeting. Unfortunately, we were not able to determine which part(s) of these 50 amino acids are critical for the sorting mechanism.

### Intracellular Localization of SLC2A9-L Deletion Mutants

**Figure 9:**
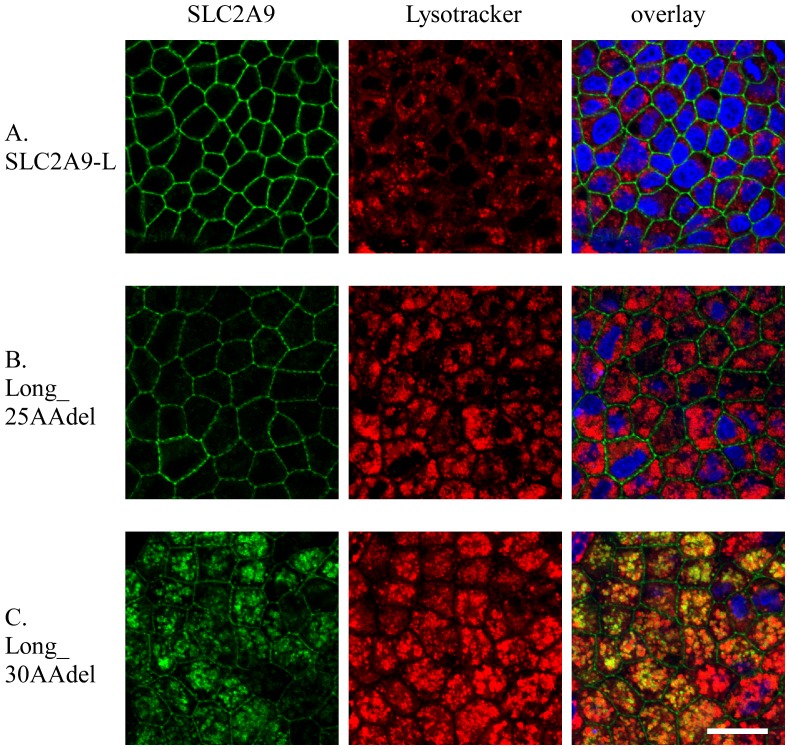
Intracellular localization of SLC2A9 deletions. GFP-tagged UARTv1-L wild-type (A), Long_25AAdel (B) and Long_30AAdel (C) were expressed in MDCK cells. These constructs were visualized in green. Lysosome of the MDCK cell was stained with lysotracker in red. Confocal images of xy obtained and the white scale bar was 20 µm.

We noticed that SLC2A9-L mutants with large deletion in the N-terminal amino acids were not only targeted to both apical and basolateral membranes, but also localized in the intracellular compartment. To identify which compartment they were expressed, MDCK cells expressing SLC2A9-L, Long_25AAdel or Long_30AAdel, were stained with intracellular organellar markers, calnexin (ER), golgin 97 (Golgi) and lysotracker (lysosome). Almost all SLC2A9-L and Long_25AAdel were localized at the basolateral cell membrane without co-localizing with any intracellular markers tested (Fig. 9A and B). In contrast, Long_30AAdel, which went to the intracellular compartment, was co-stained with lysotracker (Fig. 9C).

## Discussion

In this study, isoform-specific antibodies against SLC2A9 were successfully developed. Using these antibodies we found that SLC2A9-L was expressed at the basolateral membrane of the kidney proximal tubular cell in humans, likely to be responsible for urate reabsorption. In addition, SLC2A9-S was expressed at the apical membrane of the kidney collecting duct cell and both isoforms were occasionally found in the endothelial cell. SLC2A9s in the endothelial cell may act as sugar uptake transporter. However, mouse systemic knockouts of *SLC2A9* do not show any obvious vascular phenotype [20]. Role of the short isoform in the collecting duct is also elusive. In healthy individuals, collecting duct cells should not see any sugar molecules in urine. Thus, SLC2A9-S in the collecting duct is unlikely to act as a sugar transporter. Although the direction of electrical potential is unfavorable, it may be able to take up urate if urinary concentration of urate becomes sufficiently high due to free water reabsorption in the collecting duct. In this case, how urate moves from the cell to the interstitial space and blood remains to be elucidated.

We then attempted to explain differential sorting of 2 isoforms of SLC2A9. Since the difference between SLC2A9-L and SLC2A9-S lies only at their N-terminus we hypothesized that basolateral sorting signal should be in the N-terminal amino acid residues.

**Figure 10:**
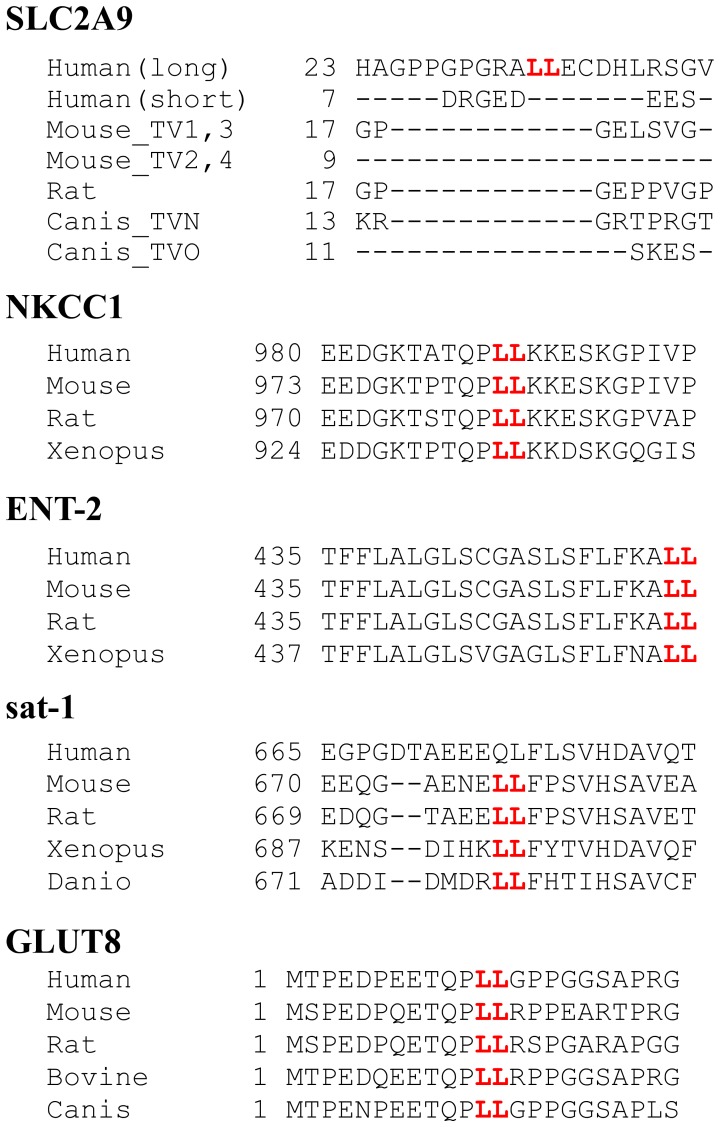
Comparison of interspecies amino acid sequences around putative LL motif in various transporters. LL in SLC2A9 was not conserved across species. LL in NKCC1, ENT2, sat-1, GLUT8 (SLC2A8) has been shown to be important in trafficking of these transporters (see text) and amino acid sequences surrounding LL were conserved.

At the beginning, we focused on putative di-leucine motif in SLC2A9-L because the motif is critical for basolateral sorting of many membrane proteins. For example, di-leucine motifs of Na-K-Cl cotransporter (NKCC) [21], nucleoside transporter [22], sulfate anion transporter [23], and SLC2A8/GLUT8 [24], [25] are critical for their basolateral trafficking. There are two NKCC isoforms, NKCC1 and NKCC2. The renal NKCC2, which does not have di-leucine motif, is selectively expressed in the apical membranes of cells of the kidney tubular cell while NKCC1, which have di-leucine motif at its C-terminus, is localized in the basolateral membranes of many epithelia. Removal of di-leucine motif from NKCC1 leads to miss-localization to apical membrane in MDCK cells [21]. Nucleoside transporter, ENT2, has di-leucine motif at the C-terminus and was localized at basolateral membrane in MDCK cells [22]. When the di-leucine motif was mutated with alanine or deleted from ENT2, surface expression was drastically reduced. There are di-leucine motif and PDZ domain in the C-terminal of sulfate anion transporter, sat-1 and it was targeted to the basolateral membrane of MDCK cells and porcine proximal tubular cells, LLC-PK1 [23]. Removal of PDZ domain from sat-1 had no effect on its localization. In contrast, conversion of the di-leucine motif in rat sat-1 at position 677/678 to alanines failed to reach the transporter to the cell membrane. GLUT8, a transporter encoded by *SLC2A8*, belongs to the same glucose transporter family as SLC2A9, has an N-terminal di-leucine motif. When GLUT8 is expressed in rat adipose cells, COS cells or HeLa cells, it exists in intracellular vesicles. In contrast, a mutant GLUT8 where the di-leucine motif is replaced with alanines is predominantly targeted to the plasma membrane [24], [25]. We also confirmed these results in our MDCK cells; Wild-type GLUT8 was localized at the intracellular compartment whereas a mutant GLUT8 at the di-leucine motif was expressed at the apical and basolateral cell surface (Kimura, unpublished observations). Thus, di-leucine motif is important for targeting of many transporters. However, as shown in Fig. 6, LL sequence in the N-terminus of SLC2A9 did not act as other di-leucine motifs mentioned above. Recently while we were preparing this manuscript, another group also reported that substitution of the LL sequence in SLC2A9-L does not affect its localization in MDCK cells [19]. In comparative genetics, it is often said that functionally important residues are likely to be conserved across species. When we compared the amino acid sequences of aforementioned transporter orthologs where the LL motif has been shown to be important in sorting, the LL motif and its neighborhood were conserved across species (Fig. 10). In contrast, the LL sequence and its surrounding residues in SLC2A9-L were not conserved (Fig. 10), suggesting that the LL sequence in SLC2A9 may not be functionally important.

Since putative di-leucine motif in SLC2A9 turned out not to be critical for its basolateral sorting, deletion constructs were used to figure out which part of SLC2A9 N-terminus is important for its targeting. Removal of the N-terminal 30 amino acids, but not the N-terminal 25 amino acids, from SLC2A9-L resulted in both apical and basolateral expression (Fig. 7A and B) and removal between 25^th^ and 50^th^ amino acids, but not between 30^th^ and 50^th^ amino acids, from SLC2A9-L leaded to both apical and basolateral expression (Fig. 8). To our surprise, replacement or deletion of the N-terminal 25^th^ to 30^th^ amino acids of SLC2A9-L did not affect its basolateral localization (Fig. 7C and D). The fact that more than 25–30 amino acid deletion from the N-terminus of SLC2A9-L disrupted its basolateral localization suggests that the N-terminus unique to this isoform is playing a role in basolateral sorting. Bibee et al concluded that the N-terminus of SLC2A9-L does not contain basolateral signal based on their result that a chimeric protein where N-terminus of GLUT3, an apically targeted protein, is substituted with that of SLC2A9-L goes to apical as wild type GLUT3 [19]. This was not apparently consistent with ours, but direct comparison may not be justifiable in view of different experimental methods; we made deletion and substitution construct of SLC2A9-L while Bibee et al made chimeric proteins. It is of note that when a mutant was targeted to both apical and basolateral membranes, it also ended up in the lysosome (Fig. 9), probably to be degraded. When 20 amino acids out of 21 unique N-terminal amino acids were removed from SLC2A9-S, the mutant was not expressed. These results raise the possibility that the N-terminal amino acids sequences are important in protein stability as well as trafficking. Unfortunately, we were not able to pin down the critical amino acid residues for basolateral trafficking of SLC2A9-L in its N-terminus. Since attempt to identify proteins interacting with the N-terminal region also failed, it is impossible for us to propose a mechanism of basolateral sorting of SLC2A9-L.

In conclusion, we found that 2 isoforms of SLC2A9 have unique intra tissue distribution in the human kidney and that the N-terminal amino acids unique to each isoform plays a role in their membrane trafficking and stability.

## Materials and Methods

### Ethics Statement

This study was carried out in strict accordance with the recommendations in the Guide for the Care and Use of Laboratory Animals of Ministry of Education, Culture, Sports, Science and Technology-JAPAN. The protocol was approved by Institute of Laboratory Animals, Graduate School of Medicine, Kyorin University (The Experimental Animal Ethics Committee in Kyorin University, permit number 90).

### Antibodies

To generate antibodies which would detect SLC2A9-S and SLC2A9-L differently, we used N-termini of SLC2A9, which have different amino acid sequences (Fig. 1A), as immunogens. pGEX constructs including the different segment of SLC2A9s were transformed into E.coli BL-21. The expression of GST fusion protein was induced with 0.1 mM IPTG and a protein extract was prepared with lysozyme treatment, freeze-thaw cycle and sonication in PBS. The extract was incubated with Glutathione Sepharose™ 4B beads (GE Healthcare Bio-Sciences Corp. Piscataway, NJ) for 6 hrs at 4°C. After incubation, these beads were washed 4 times with PBS and eluted with glutathione elute buffer (50 mM Tris-HCl, 10 mM Reduced Glutathione, pH 8.0). Eluted proteins were dialyzed with PBS and injected into rabbits together with TiterMax Gold Adjuvant (Sigma-Aldrich, St. Louis, MO). After 5th injection of the antigen, serum was collected.

### Plasmid Construction

cDNAs of SLC2A9-S and SLC2A9-L were purchased from OriGene Technologies (Rockville, MD) and Fisher Scientific (Pittsburgh PA), respectively. To construct GFP fusion proteins, SLC2A9s were amplified by polymerase chain reaction (PCR). This construct was subcloned as a EcoRI/KpnI fragment into the pEGFP-N2 vector (Clontech Laboratories, Mountain View, CA), in which the insert was fused to the amino terminus of GFP. To construct GST fusion proteins for producing antibodies, SLC2A9s were amplified by PCR with primers that included EcoRI and XhoI restriction sites. The PCR fragment was subcloned into pGEX-6P-1 vector, in which the insert was fused to the carboxyl terminus of glutathione S-transferase. All PCR primer sequences are available on request.

### Cell Culture and Transfection

COS-7 cells and MDCK cells (American Type Culture Collection (ATCC), Manassas, VA) were cultured in a humidified incubator under 5% CO_2_ in Dulbecco’s Modified Eagle’s Medium (Sigma-Aldrich, St. Louis, MO) supplemented with 10% FBS, 2 mM L-glutamine, 50 U/mL penicillin and 50 µg/ml streptomycin. DNA transfection into COS cells or MDCK cells was performed with Lipofectamine 2000 (Life Technologies, Carlsbad, CA) according to the manufacturer’s instructions. Two days after transfection, following assays were performed.

### Antibody Reactivity Checking

Reactivity of produced antibodies were examined by Western blotting and immunofluorescence. For Western blotting analysis, transfected COS cells were incubated with lysis buffer containing 1% Triton X-100, 150 mM NaCl, 5 mM EDTA, and 25 mM Tris-HCl, pH 7.4 for 30 min at 4°C. Insoluble material was removed through centrifugation at 10,000 g for 30 min at 4°C. After centrifugation, 30µg of protein were mixed with SDS-PAGE sample buffer. The samples were separated by SDS-PAGE and analyzed by Western blotting. For antigen pre-absorption test, antibodies were incubated with GST fusion proteins and Glutathione Sepharose™ 4B beads (GE Healthcare Bio-Sciences Corp. Piscataway, NJ). Overnight incubation at 4°C, supernatant was used for Western blotting. For immunofluorescence, COS cells were seeded on cover slips before transfection. Two days after transfection, cells were fixed with cold methanol and immunofluorescence was performed with anti-SLC2A9 antibodies as shown in *immunohistochemistry* part.

### Immunohistochemistry

To examine the distribution of SLC2A9 within the kidney, human tissue sections purchased from BioChain Institute, Inc. (Newark, CA) were stained with TSA Fluorescence System (PerkinElmer, Inc., Waltham, MA) according to the manufacturer’s instructions with minor modifications [26]. In brief, sections were blocked with TNB buffer (0.1 M Tris-HCl, pH 7.5, 0.15 M NaCl and 0.5% Blocking Reagent) containing 5% normal donkey serum. To block endogenous biotin, the sections were further treated with the Avidin–Biotin block (Vector Laboratories, Burlingame, CA), and endogenous peroxidase activity was quenched with 1% H_2_O_2_. The primary antibody was applied overnight at 4°C. Slides were washed with TNT Wash Buffer (0.1 M Tris-HCl, pH 7.5, 0.15 M NaCl, and 0.05% Tween20) and incubated with biotin-conjugated donkey anti–rabbit IgG (Jackson ImmunoResearch Laboratories, Inc., West Grove, PA), followed by incubation with streptavidin–HRP conjugate (Life Technologies, Carlsbad, CA) together with 1 µg/ml DAPI and 10 µg/ml fluorescein-Lotus Tetragonolobus lectin or 10 µg/ml rhodamine-Dolichos Biflorus agglutinin (Life Technologies, Carlsbad, CA). The antigens were detected using tyramide-FITC or tyramide-Cy3. Sections were mounted in Fluorescence Mounting Medium (Dako, Glostrup, Denmark). Fluorescence was visualized with an Olympus Fluoview FV1000 laser confocal microscope. Images are the product of 3-fold line averaging. Contrast and brightness settings were chosen so that all pixels were within the linear range.

### Cell Surface Biotinylation

Two days after transfection, MDCK cells were pated on the cell culture inserts (BD, Franklin Lake, NJ) in the presence of 1 mg/ml G418 and were grown to confluence. Cells were washed in ice-cold PBS^++^ (PBS supplemented with 0.1 mM CaCl_2_ and 1 mM MgCl_2_) and incubated with 2.5 mM Sulfo-NHS-SS-biotin (Thermo Fisher Scientific, Rockford, IL) in PBS^++^ for 30 min. Excess biotin was quenched 3×5 min with 100 mM glycine in PBS^++^. Cell surface membrane was visualized with Alexa Fluor® 594 conjugated streptavidin (Life Technologies, Carlsbad, CA). Cells were mounted in Fluorescence Mounting Medium (Dako, Glostrup, Denmark). Fluorescence was visualized with an Olympus Fluoview FV1000 laser confocal microscope. Images are the product of 3-fold line averaging. Contrast and brightness settings were chosen so that all pixels were within the linear range.

### Staining of Intracellular Organelle

Two days after transfection, MDCK cells were pated on the cell culture inserts (BD, Franklin Lake, NJ) in the presence of 1 mg/ml G418 and were grown to confluence on the cell culture inserts. Cells were incubated with 75 nM LysoTracker (Life Technologies, Carlsbad, CA), in growing medium for 2 hrs. Samples were washed 3 times with PBS^++^ (PBS supplemented with 0.1 mM CaCl_2_ and 1 mM MgCl_2_) and fixed with 4% paraformaldehyde, stained with DAPI. Cells were washed with PBS and mounted in Fluorescence Mounting Medium (Dako, Glostrup, Denmark). For detection of ER and Golgi, transfected MDCK cells were fixed with cold methanol and immunofluorescence was performed with anti-calnexin antibody (Enzo Life Sciences, Farmingdale, NY) or anti-golgin97 antibody (BD Biosciences, San Jose, CA) as shown in *immunohistochemistry* part.

## References

[1] Kamatani Y, Matsuda K, Okada Y, Kubo M, Hosono N, et al (2010) Genome-wide association study of hematological and biochemical traits in a Japanese population. Nat Genet 42: 210–215. 20139978 10.1038/ng.531

[2] Kolz M, Johnson T, Sanna S, Teumer A, Vitart V, et al (2009) Meta-analysis of 28,141 individuals identifies common variants within five new loci that influence uric acid concentrations. PLoS Genet 5: e1000504. 19503597 10.1371/journal.pgen.1000504 PMC2683940

[3] Kottgen A, Albrecht E, Teumer A, Vitart V, Krumsiek J, et al (2013) Genome-wide association analyses identify 18 new loci associated with serum urate concentrations. Nat Genet 45: 145–154. 23263486 10.1038/ng.2500 PMC3663712

[4] Li S, Sanna S, Maschio A, Busonero F, Usala G, et al (2007) The GLUT9 gene is associated with serum uric acid levels in Sardinia and Chianti cohorts. PLoS Genet 3: e194. 17997608 10.1371/journal.pgen.0030194 PMC2065883

[5] Vitart V, Rudan I, Hayward C, Gray NK, Floyd J, et al (2008) SLC2A9 is a newly identified urate transporter influencing serum urate concentration, urate excretion and gout. Nat Genet 40: 437–442. 18327257 10.1038/ng.106

[6] Phay JE, Hussain HB, Moley JF (2000) Cloning and expression analysis of a novel member of the facilitative glucose transporter family, SLC2A9 (GLUT9). Genomics 66: 217–220. 10860667 10.1006/geno.2000.6195

[7] Augustin R, Carayannopoulos MO, Dowd LO, Phay JE, Moley JF, et al (2004) Identification and characterization of human glucose transporter-like protein-9 (GLUT9): alternative splicing alters trafficking. J Biol Chem 279: 16229–16236. 14739288 10.1074/jbc.M312226200

[8] Anzai N, Ichida K, Jutabha P, Kimura T, Babu E, et al (2008) Plasma urate level is directly regulated by a voltage-driven urate efflux transporter URATv1 (SLC2A9) in humans. J Biol Chem 283: 26834–26838. 18701466 10.1074/jbc.C800156200

[9] Caulfield MJ, Munroe PB, O’Neill D, Witkowska K, Charchar FJ, et al (2008) SLC2A9 is a high-capacity urate transporter in humans. PLoS Med 5: e197. 18842065 10.1371/journal.pmed.0050197 PMC2561076

[10] Matsuo H, Chiba T, Nagamori S, Nakayama A, Domoto H, et al (2008) Mutations in glucose transporter 9 gene SLC2A9 cause renal hypouricemia. Am J Hum Genet 83: 744–751. 19026395 10.1016/j.ajhg.2008.11.001 PMC2668068

[11] Dinour D, Gray NK, Campbell S, Shu X, Sawyer L, et al (2010) Homozygous SLC2A9 mutations cause severe renal hypouricemia. J Am Soc Nephrol 21: 64–72. 19926891 10.1681/ASN.2009040406 PMC2799278

[12] Dinour D, Gray NK, Ganon L, Knox AJ, Shalev H, et al (2012) Two novel homozygous SLC2A9 mutations cause renal hypouricemia type 2. Nephrol Dial Transplant 27: 1035–1041. 21810765 10.1093/ndt/gfr419

[13] Stiburkova B, Ichida K, Sebesta I (2011) Novel homozygous insertion in SLC2A9 gene caused renal hypouricemia. Mol Genet Metab 102: 430–435. 21256783 10.1016/j.ymgme.2010.12.016

[14] Stiburkova B, Taylor J, Marinaki AM, Sebesta I (2012) Acute kidney injury in two children caused by renal hypouricaemia type 2. Pediatr Nephrol 27: 1411–1415. 22527535 10.1007/s00467-012-2174-0

[15] Enomoto A, Kimura H, Chairoungdua A, Shigeta Y, Jutabha P, et al (2002) Molecular identification of a renal urate anion exchanger that regulates blood urate levels. Nature 417: 447–452. 12024214 10.1038/nature742

[16] Kimura T, Amonpatumrat S, Tsukada A, Fukutomi T, Jutabha P, et al (2011) Increased expression of SLC2A9 decreases urate excretion from the kidney. Nucleosides Nucleotides Nucleic Acids 30: 1295–1301. 22132990 10.1080/15257770.2011.628354

[17] Manolescu AR, Augustin R, Moley K, Cheeseman C (2007) A highly conserved hydrophobic motif in the exofacial vestibule of fructose transporting SLC2A proteins acts as a critical determinant of their substrate selectivity. Mol Membr Biol 24: 455–463. 17710649 10.1080/09687680701298143

[18] Witkowska K, Smith KM, Yao SY, Ng AM, O’Neill D, et al (2012) Human SLC2A9a and SLC2A9b isoforms mediate electrogenic transport of urate with different characteristics in the presence of hexoses. Am J Physiol Renal Physiol 303: F527–539. 22647630 10.1152/ajprenal.00134.2012 PMC3423118

[19] Bibee KP, Augustin R, Gazit V, Moley KH (2013) The apical sorting signal for human GLUT9b resides in the N-terminus. Mol Cell Biochem 376: 163–173. 23361362 10.1007/s11010-013-1564-3 PMC5028210

[20] Preitner F, Bonny O, Laverriere A, Rotman S, Firsov D, et al (2009) Glut9 is a major regulator of urate homeostasis and its genetic inactivation induces hyperuricosuria and urate nephropathy. Proc Natl Acad Sci U S A 106: 15501–15506. 19706426 10.1073/pnas.0904411106 PMC2741280

[21] Carmosino M, Gimenez I, Caplan M, Forbush B (2008) Exon loss accounts for differential sorting of Na-K-Cl cotransporters in polarized epithelial cells. Mol Biol Cell 19: 4341–4351. 18667527 10.1091/mbc.E08-05-0478 PMC2555935

[22] Mangravite LM, Xiao G, Giacomini KM (2003) Localization of human equilibrative nucleoside transporters, hENT1 and hENT2, in renal epithelial cells. Am J Physiol Renal Physiol 284: F902–910. 12527552 10.1152/ajprenal.00215.2002

[23] Regeer RR, Markovich D (2004) A dileucine motif targets the sulfate anion transporter sat-1 to the basolateral membrane in renal cell lines. Am J Physiol Cell Physiol 287: C365–372. 15070814 10.1152/ajpcell.00502.2003

[24] Lisinski I, Schurmann A, Joost HG, Cushman SW, Al-Hasani H (2001) Targeting of GLUT6 (formerly GLUT9) and GLUT8 in rat adipose cells. Biochem J 358: 517–522. 11513753 10.1042/0264-6021:3580517 PMC1222087

[25] Schmidt U, Briese S, Leicht K, Schurmann A, Joost HG, et al (2006) Endocytosis of the glucose transporter GLUT8 is mediated by interaction of a dileucine motif with the beta2-adaptin subunit of the AP-2 adaptor complex. J Cell Sci 119: 2321–2331. 16723738 10.1242/jcs.02943

[26] Zhao X, Deak E, Soderberg K, Linehan M, Spezzano D, et al (2003) Vaginal submucosal dendritic cells, but not Langerhans cells, induce protective Th1 responses to herpes simplex virus-2. J Exp Med 197: 153–162. 12538655 10.1084/jem.20021109 PMC2193810

